# Boundaries as taboo: a cultural-psychological model of human-nature relations in Japanese heterogeneous marriage narratives

**DOI:** 10.3389/fpsyg.2026.1815276

**Published:** 2026-04-24

**Authors:** Jianhua Liu

**Affiliations:** Department of Japanese, School of Foreign Languages, Jiangnan University, Wuxi, China

**Keywords:** cultural schema, human-nature relations, Japanese folklore, moral emotions, narrative psychology, psychological boundaries, taboo

## Abstract

Human cognition of the relationship among individuals, nature and culture is influenced not only by material conditions but also by culturally inherited ways of perceiving and interpreting the world. Among the many ways in which such orientations are passed on, narrative traditions have persisted for an extended period. However, in the past folklore researches, the way that such narrative structures encode shared psychological orientations towards nature has seldom been explored. This study was based on Japanese Heterogeneous Marriage Narratives and explains the recurring taboo motifs in them as cultural means of marking the boundaries between humans and nature. In light of the ideas from narrative psychology and cultural psychology, it will be explored how such stories regulate emotional orientation toward nature. Through qualitative comparison of representative narratives from classical texts and folktales, attention was paid to prohibitions such as “do not look” and “do not open,” and these taboos are not regarded merely as plot devices, but rather as ways of regulating how people approach, fear, or distance themselves from the non-human world, thus shaping perception and behavior. The results reflect the recurring features of the construction and perception of boundaries in multiple narrative contexts. Based on this, the article puts forward a three-stage cultural-psychological model —Intimate, Transitional, and Defensive—which reflects different modes of boundary cognition, ranging from relatively close coexistence with nature to increasing separation and exclusion. Through the combined application of literary-anthropological materials and psychological theories, this study shows that traditional narratives carry lasting natural common concepts and continue to influence contemporary people’s environmental cognition. These narratives provide an important cultural and psychological form for understanding, sorting out and explaining the collective experience of human beings in dealing with the changes of the earth’s system and related human collective trauma. Therefore, its value in future sustainable development research and environmental education cannot be ignored.

## 1 Introduction

In recent years, growing ecological concerns have prompted renewed attention to how humans conceptualize their relationship with the natural world. Many people’s discussions of the sustainability of development are mainly focused on science and technology, policies and regulations, or economic systems (Braito et al., 2017; Lobler, 2017; Lengieza et al., 2023), But these external measures were based on the basic habits of perception and feeling. Whether nature is considered to be relatives, a sacred object, a useful resource or an impending danger, after prolonged integration with human life over many years and in decisions involving groups of people throughout all aspects of existence for a long time, such perceptions have now become closely linked. These orientations are not formed solely by material conditions. They are also transmitted via culturally shared ways of understanding the world, many of which are carried forward and preserved through stories.

Narrative forms also have a lasting effect in this process. Stories serve to tell events and amuse people, but they also organize experience. According to Bruner, stories are one of the ways people think; they differ from logical inference and paradigmatic reasoning in that they help organize one’s life experiences and gain an understanding thereof. Recurrent plots and images can help us discover the patterns of Agency, a sense of responsibility, and ownership (Bruner, 1990). In this way, myths and folktales can be read not only as literary works but also as carriers of collectively inherited cognitive and affective patterns about society and nature.

Cultural psychology believes that these trends stem from a system of values and interpretations accumulated over an extended period. Cognition and emotion are formed in the local “moral world” of shared moral worlds, rather than emerging from an individual process (Shweder, 1991). Classical anthropological discussions of taboos suggest that these distinctions are often maintained by prohibition. Douglas (2003) once said that pollution is “matter out of place”, and thus taboo serves as a means of protection for symbolic classifications and the integrity of category boundaries. Therefore, prohibitions are not only a restriction on behavior but also the formation of patterned emotional reactions, that is, reverence for what is perceived as sacred, caution towards uncertainty, and avoidance of danger or pollution.

Research on moral emotions helps us understand the operation of this kind of response in practice. Feelings of awe tend to arise when facing something vast or powerful, and they promote a respectful distance and accommodation (Keltner and Haidt, 2003). Disgust, however, which functions to avoid contamination, may lead to rejection or exclusion (Rozin et al., 2000). In this sense, emotions operate as regulatory mechanism to determine how close people feel they can get to the non-human world. In summary, from various angles, it can be concluded that the cognition boundaries of relationships between people and nature have been gradually formed through narrative taboos and emotions.

These questions, however, have rarely been addressed within psychological research using traditional narrative materials. In Japan, Yanagita (1985)’s collections of folktales and legends have preserved many stories of animal wives, spirit spouses, and encounters with other-than-human beings; these are treated as records of local cosmology and everyday belief. Orikuchi (1975) also interpreted such narratives in connection with visits by beings of another world and the thin line separating the human community from the sacred. Using comparative and structural methods can help us better understand the patterned forms of these stories. Propp (1968)‘s morphological analysis found a recurring sequence of interdiction, violation and consequence—A structure similar to “do not look” or “do not open,” which can be frequently observed in many stories of heterogeneous marriage. Taking into account the wider field of anthropology, according to Levi-Strauss (2008), myths are expressions of underlying oppositions, such as nature and culture, and marriage and exchange symbolically negotiate these category differences. Together, this body of research has provided detailed historical and textual foundations for the narrative and symbol system of such stories.

Despite these contributions, previous research has primarily treated such narratives as symbolic or literary forms, with limited attention to their potential role in organizing shared psychological orientations—particularly in relation to emotional responses and perceived proximity to the non-human world. On the other hand, psychological research on narrative, culture and environmental attitudes has mainly been carried out through contemporary interviews, surveys or experiments, and traditional narrative corpora have seldom been utilized as sources of collective meaning. As a result, the longer cultural histories through which emotional orientations towards nature are formed have not been adequately investigated.

The present study seeks to address this gap by integrating narrative psychology and cultural psychology. It shows that the taboo images in Japanese heterogeneous marriage stories serve as cultural tools to regulate boundaries among Humans and non-Human Beings. Based on the integration of literary-anthropological materials and psychological theory, this study seeks to clarify how narrative traditions contribute to shape emotional orientations toward the environment.

## 2 Theoretical framework

### 2.1 Cultural schemas as shared cognitive–affective systems

This study adopted a cultural schema perspective within cultural psychology as its central theoretical framework. Bartlett (1995)’s notion of the schema first came into being as he saw that there are two modes by which people acquire knowledge: recollection or reconstruction and association, both require organisation to become a stable cognitive element. Therefore, schemas are used to build the background for perception and understanding.

Within cultural psychology, this idea has expanded to highlight the collective and socially mediated aspects of culture. Shweder (1991) defines culture as consisting of “shared meanings” that organise the psychological processes; Wertsch (1998) also posits that culture objects serve as catalysts for cognition. In terms of cognition, the scheme itself is also a unit of memory, and at this time some meaning has already been constructed in one’s mind.

Cultural Schemas are not only cognitive processes, but also emotional components attached to it. According to Strauss and Quinn (1997), schemas are “networks of closely linked components” that not only encompass emotions but also a range of other associations. That is, cultural knowledge not only includes the things that have been learned but also the emotions or feelings that people experience in certain situations.

Further to this, cultural schemas refer to sets of classifications used to organise the different kinds of being. Mary Douglas’ work (1966/2003) can be cited as an example here. Douglas suggests that cultural systems are based on a classification rule for separating purity and impurity or out-of-place things. These classifications help to maintain the symbol of order. When applied to human-non-human relations, these classification systems establish the domains of being in existence and standards for determining violations.

Therefore, cultural schemas both organise cognition and define the symbols’ boundaries as well as establish normative expectations. The base that underpins their analysis of traditional narratives as manifestations of cultural-linguistic psychological orientation clusters.

### 2.2 Narrative psychology and the organization of experience

Considering that it is organised in terms of expressing and passing on cultural concepts, this paper applies narrative psychology for analysis. Based on Bruner’s view (1990) that narrative constitutes the means of organising human experiences into meaning, they are primary means for presenting the organisation of perceptions of events rather than second-order representations.

Narratives organise experience by connecting actions, intentions and outcomes in a sequential chain. Generally, stories include a deviation from the conventional expectation and then have some kind of resolution; People can understand these deviations through cultural consensus. Thus, in some sense, narrative form is inherently associated with its role in regulating people’s expectations and interpreting transgressions.

As for the folklore, stories can be regarded as culturalized models to repeat encoding a particular form of interaction system. Recurrences of the prohibition-violation-outcome cycle found in many human-nonhuman marriage stories are thus characteristic of it. In this way, it aligns with the structure analysis by Propp (1968). Although this study has a certain distance from the structure theory framework; these typical cases can be seen as paths to organise cultural experience.

Narrative psychology points out that stories can help people link their own thoughts with cultural understanding through mediation. By repeatedly encountering culturally shared stories, people develop a sense of expectation and interpretation through these narratives. Narratives serve as vehicles for transmitting cultural schemas, encoding cultural meanings in explicit or implicit experiential sequences.

### 2.3 Moral emotions as mechanisms of boundary regulation

Culture-Schema Theory organizes shared meaning and Narrative Psychology accounts for its organised expression; however, an additional theoretical perspective needs to be added in order to explain how these meanings affect perception and behavior psychologically. Therefore, in this study, we draw on theories of moral emotions.

Moral emotions refer to affective reactions associated with judgments of actions relative to cultural standards. According to Haidt (2001), moral emotions have an essential function of guiding people’s behavior through instant, embodied judgments of morality versus immorality. These moral emotions include various emotions such as fear, shame, guilt, disgust, and anger under different kinds of moral judgment.

Morality, as a component of culture. Although they cannot be regarded as universally human responses, their formation depends on cultural expectations and group classification. Therefore, in light of the concepts of purity and pollution first put forward by Douglas (2003), this paper will adopt them for analysis. Based on Douglas’ analysis, these feelings of pollution result from deficiencies in the system’s category criteria. Emotions bring about feelings of protection over the symbolic structure by individuals in society.

Within this framework, prohibitions function to stimulate anticipatory moral sentiments among audiences. They could be fear of overstepping the mark or an awareness not to go too far. When violations happen, people are more likely to feel stronger emotions of fear, shame and disgust. These emotional reactions are not incidental; instead, they constitute the primary mechanism through which cultural norms are enforced.

Emotional regulation of narrative conclusions such as separation, disappearance and irreversible changes can then be explained. Strengthen the connection between transgression and negative emotions in order to uphold cultural propriety. In this sense, moral emotions, as a regulating force, closely link cultural patterns with physical experience.

### 2.4 Integrating narrative structure, cultural schemas, and moral emotions

Combining cultural schema theory, narrative psychology and moral emotion to form an in-depth analysis system of human-nature marriage narratives from psychological-cultural perspectives is provided. Each part has its own contribution but works together to form a whole; narrative psychology provides structural explanations, cultural schema theory offers shared system of meaning, and moral emotions describe regulatory mechanisms.

Recently, some cultural-psychological-oriented studies have reached the same conclusion. According to Mesquita et al. (2016), emotional experiences are generated by the interaction of cultural meaning and situation background. Valsiner (2014) also thinks that meaning construction is a dynamic process involving the continuous interaction between cultural systems and individual experiences.

Therefore, the recurring pattern of prohibition–violation–outcome reflects not only the narrative form, but also a cognitive-emotional process constructed through culture.

Narratives can be defined in terms of cultural-cognized schemata that are not only cognitively organised but also imbued with an emotional charge. Recurrent patterns of prohibition, violation and outcomes indicate a scheme to organise expectations regarding the right boundaries for interactions among human beings and non-humans. Not only cognitive boundaries, but also symbols that constitute a system of classification based on these divisions.

Moral emotion serves to connect each stage of the story plot and express diverse emotions through it. Prohibitions will evoke the expected emotion of maintaining boundary stability; potential violations will cause emotional tension; and transgressions will trigger a strong emotional reaction and eventually lead to narrative resolution. The process reflects the interaction between cognitive structure, narrative form and emotional mechanism.

### 2.5 Boundary cognition as an integrated cultural–psychological process

Based on this theoretical integration, in order to further clarify the concept of heterogeneous marriage narratives as cultural resources for constructing boundary cognition. Boundary cognition is the way people recognize, judge and respond to differences in categorisation among different entities of existence, especially humans and non-humans.

Boundary cognition is a result of schema, narrative and emotion interactions within this scheme. Culture schemata constitute the underlying classification system; Narrative encodes these schemata as experiential forms; and moral emotions make them psychologically significant through the link with affective response. This forms a stable expectation and evaluation model at the cultural level.

Analytical dimensions employed in this study directly originate from this framework: Prohibition, Violation, Emotional Tone, and Narrative Outcome. Prohibition corresponds to the expectation based on the classification system and the scheme; Violation represents a disruption of the symbolized order; Emotion reflects the activation of moral emotion; and outcome shows boundary repair or reorganization.

## 3 Materials and methods

### 3.1 Research design

This research adopted qualitative and theory-oriented narrative analysis. Instead of using statistical measurement to identify the recurrence pattern among cases by comparing and coding systematically. Hence, the conventional account is understood to be culturally-captured psychological matter with a generally fixed cognition-emotion configuration.

Narrative inquiry in psychology has established stories as legitimate analytical data. Rather than considering variables as independent objects, narrative research focuses on the organization of experience in time-ordered stories that carry culturally shared values and expectations (Polkinghorne, 1995; Riessman, 2008). In this case, the material is more likely to have embedded norms and boundaries within the plot structure rather than in an explicit form.

Accordingly, the present study does not attempt to verify the historical origins of particular tales. Instead, it examines how recurring narrative devices—especially prohibitions and their violation—systematically organise perceptions of acceptable and unacceptable contact between humans and non-human beings.

### 3.2 Materials

The corpus analyzed in this study consists of twelve Japanese human–nonhuman marriage narratives drawn from authoritative folklore and literary sources that preserve both classical and orally transmitted traditions. These materials include early myth-historical texts [e.g., *Kojiki* (Ono, 1982), *Nihon shoki* (Sakamoto et al., 1994), Fudoki (n.d.), *Manyoushyu* (Otomo, 2009), *Nihon Ryoiki* (Keikai, 1975)], medieval narrative collection *Konjaku Monogatarishu* (Tyler, 2012) and *Okagami* (McCullough, 1980), and modern ethnographic and regional folklore compilations produced within the Japanese folklore tradition, such as local tale archives and prefectural folk records (Orikuchi, 1995). Together, these sources represent the most widely cited documentary foundations of Japanese vernacular storytelling and have long served as primary materials in folklore and religious studies research. The purpose of this corpus is analytical generalization rather than statistical representativeness.

To enable systematic comparison, the narratives were divided into two structurally parallel groups: heterogeneous wife stories (Table 1) and heterogeneous husband stories (Table 2). The wife group includes well-known motifs such as the crane wife, fox wife, snake/dragon bride, and other animal or spirit women who marry human men. The husband group comprises serpent, monkey or other nonhuman male spouses who enter marital relationships with human women. This gender-based division reflects a long-standing typological distinction in Japanese folklore studies and allows for the examination of whether boundary regulation differs according to the direction of human–nonhuman union.

**Table 1:** Coding results of taboo motifs in heterogeneous wife narratives.

Source	Story	Coding unit ID	Prohibition statement	Boundary domain (P/S/B/T/R)	Violation behavior	Narrative outcome	Emotional tone	Stage
*Omi Fudoki*	Swan Maiden (Feather Robe)	W1-1	Do not reveal the hiding place of my feather robe	P, R	The male failed to keep the secret; the maiden retrieved her robe	The swan maiden returned to the celestial realm, ending the marriage	Sorrowful and regretful, no antagonism	Intimate
*Kojiki*, *Nihon Shoki*	Toyotama-hime (Crocodile Wife)	W2-1	Do not look upon me when I give birth	P	Hoori peeked and saw her true crocodile form during childbirth	Toyotama-hime returned to the sea in shame, never to return	Intertwined with shame and regret, peaceful separation	Intimate
*Fudoki*, *Nihon Shoki*, *Manyoshu*	Urashima Taro (Dragon Palace Wife)	W3-1	Do not open the tamatebako until you return home	P, S	Urashima Taro opened the sealed box out of confusion	He aged instantly and was forever separated from the dragon palace	Desolate and helpless, with melancholy over time and separation	Intimate
*Nihon Ryoiki* (Vol. 1)	Fox Wife	W4-1	Do not reveal my true form to others	P	A dog chased her, forcing her to jump on the roof and reveal her fox form	The fox wife left; their children became clan ancestors	Subtle reluctance beneath calmness, no conflict	Intimate
*Okagami*	Fox Wife (Mother of Abe no Seimei)	W5-1	Do not look upon my true form	P	Abe no Seimei accidentally saw his mother’s fox form	She departed sorrowfully, leaving spiritual powers to Seimei	Sorrowful yet consolatory, a blend of maternal love and grief	Intimate
Kagoshima folk narrative	Crane Wife (Kagoshima Version)	W6-1	Do not watch me while I weave	P	Karoku spied on her weaving with her own feathers	She lost her human form and flew back to the sky	Intertwined with regret and guilt, caused by human curiosity	Intimate

W = wife narrative; each coding unit represents one complete taboo episode within a story rather than the entire tale.

Boundary Domain (P/S/T/R) indicates the primary type of restriction: P = perceptual taboo (concerning limits on seeing, hearing, or knowing); S = spatial taboo (concerning movement, distance, or entry into restricted places); B = bodily taboo (concerning physical contact, corporeal exposure, or material interaction); T = temporal taboo (concerning timing, duration, or sequence of action); R = relational taboo (concerning social roles, obligations, or interpersonal commitments). Multiple letters indicate overlapping functions.

Violation Behavior records the concrete action that breaks the prohibition.

Narrative Outcome summarizes the immediate consequence of violation (e.g., disappearance, separation, expulsion, return to another realm).

Emotional Tone captures the dominant affective atmosphere of the separation scene (e.g., sorrowful, ambivalent, fearful), based on textual description rather than interpretive inference.

Stage refers to the boundary configuration identified through cross-case comparison: Intimate = closeness and permeability between human and non-human domains; Transitional = ambivalent coexistence marked by uncertainty and instability; Defensive = rigid separation emphasizing danger or exclusion.

Stage assignment is determined by the overall narrative logic and emotional framing of the episode rather than by the mere presence of a taboo.

**Table 2:** Coding results of taboo motifs in heterogeneous husband narratives.

Source	Story	Coding unit ID	Prohibition statement	Boundary domain (P/S/B/T/R)	Violation behavior	Narrative outcome	Emotional tone	Stage
Kojiki	Serpent Husband (Mt. Miwa, Ikutamayorihime)	H1-1	Do not follow me or track my path	P	Ikutamayori hime tracked him with hemp thread and saw his serpent form	His divine identity was revealed; they bore clan ancestors	Peaceful and relieved, no conflict or sorrow	Intimate
Nihon Shoki	Serpent Husband (Nihon Shoki, Yamato-totohi-momoso-hime)	H2-1	Do not open my dressing case	P	She opened the case, saw his serpent form, and screamed in shock	The serpent god left in insult; the wife committed suicide out of regret	Thrilling and desolate, a blend of humiliation, guilt and tragedy	Transitional
Hizen Fudoki (Matsumura District)	Serpent Husband (Hizen Fudoki, Otohime)	H3-1	Do not follow me to the marsh	P	Otohime tracked him with hemp thread and found his serpent form in the marsh	The serpent husband killed Otohime, whose body was found in the marsh	Frightening and brutal, filled with antagonism and malice	Defensive
Nihon Ryoiki (Middle Vol., Scroll 8)	Serpent Husband 1 (Nihon Ryoiki, Okisome-no-omitaime)	H4-1	Do not break our marriage covenant or resist my touch	R, B	She first agreed to marry him, then refused and resisted contact under a monk’s guidance	A crab killed the serpent; she was rescued	Tense and alert, a blend of resistance and relief, no compassion	Defensive
Nihon Ryoiki (Middle Vol., Scroll 41)	Serpent Husband 2 (Nihon Ryoiki, Wealthy Family Daughter)	H5-1	Do not resist my advances or let your family interfere	R, B	The serpent forced contact; her family intervened with medicine to resist	The family killed the serpent; the girl escaped danger	Frightened and disgusted, filled with repulsion and confrontation	Defensive
Konjaku Monogatarishu	Ape (Monkey) Husband	H6-1	Do not spy on me when I move in the forest in my ape form at midnight	P	The wife spied on her husband moving in the forest in his ape form late at night	The ape husband left his human wife and returned to the mountain after his secret was exposed	Sorrowful and alienated, with disappointment over broken trust	Transitional

H = husband narrative; each coding unit represents one complete taboo episode within a story rather than the entire tale.

Boundary Domain (P/S/T/R) indicates the primary type of restriction: P = perceptual taboo (concerning limits on seeing, hearing, or knowing); S = spatial taboo (concerning movement, distance, or entry into restricted places); B = bodily taboo (concerning physical contact, corporeal exposure, or material interaction); T = temporal taboo (concerning timing, duration, or sequence of action); R = relational taboo (concerning social roles, obligations, or interpersonal commitments). Multiple letters indicate overlapping functions.

Violation Behavior records the concrete action that breaks the prohibition.

Narrative Outcome summarizes the immediate consequence of violation (e.g., disappearance, separation, expulsion, return to another realm).

Emotional Tone captures the dominant affective atmosphere of the separation scene (e.g., sorrowful, ambivalent, fearful), based on textual description rather than interpretive inference.

Stage refers to the boundary configuration identified through cross-case comparison: Intimate = closeness and permeability between human and non-human domains; Transitional = ambivalent coexistence marked by uncertainty and instability; Defensive = rigid separation emphasizing danger or exclusion.

Stage assignment is determined by the overall narrative logic and emotional framing of the episode rather than by the mere presence of a taboo.

To maintain the openness of the corpus construction, we conducted filtering at two stages. Firstly, an extensive collection of Japanese heterogeneous-marriage stories was compiled from classic literature, folk tales and ethnography materials. Secondly, in terms of the inclusion criterion for narrations: If there is a stable relationship between humans and nonhumans; if the prohibition or taboo statements are presented explicitly; and finally, whether it constitutes an entire narrative structure that includes the sequence from violating to punishment. Narratives without any of the following elements were eliminated.

Therefore, this procedure yielded 12 stories that were not statistically representative, but theoretically focal; these could depict recurring patterns of boundary control within the cultural system.

The above indicators led to a small number of research samples. These works were not representative; rather, they occurred frequently and remained stable over time, thus being appropriate to identify the common structure of these schemas. Due to their frequent occurrences at various locations and over time, such stories can be regarded as cultural stabilized narratives templates and are therefore relatively more suitable for comparative studies of taboo types, emotional atmosphere and boundary stages. In view of this, the corpus is a sample for analysis that aims to reflect the structural regularity of imagination about human-non-human relationships.

Through the coding system of wives’ and husbands’ narratives, a foundation has been established to explore common mechanisms as well as directionally asymmetric displays of intimacy, risk, and separation across cultural taboos.

### 3.3 Analytical framework

The analysis was conducted using close reading and thematic narrative comparison. The purpose is not to count the frequency of the motives but to find the patterned meanings that recur in different cases. A series of reflexive thematic analysis procedures commonly used in Qualitative Psychology are followed to form themes through several rounds of iteration and interaction with the data rather than by adopting pre-set coding framework (Braun and Clarke, 2006).

The coding categories were derived deductively from the theoretical framework, linking narrative structure to narrative psychology, boundary representation to cultural schema theory, and emotional orientation to moral emotion theory.

Three analytic dimensions guided the reading of each text:

First, the narrative structure: using morphological and structural methods of folktales, attention was paid to the repeated sequences such as interdiction, violation, and separation. Among these, several positions were deemed to contain the narrative boundary regulation explicitly.
Second, boundary representation: Episode examination of how it defines or destabilizes the distinction between humans and non-humans, such as concealment of bodily form, spatial separation, or restrictions on vision and knowledge.
Third, emotional orientation: Descriptions of characters’ responses and the implied audience reaction are considered to be culturally relevant emotions such as awe, fear and disgust. They can be referred to as psychological distance created by people’s perceptions of objects or events, etc.

Three analytical dimensions used in this paper are based on the theoretical foundation established in Section 2 of integration. The narrative structure is a reflection of temporal organization in experience; the boundary representation can be found in cultural schema theory that explores shared systems for classification and boundary-setting; Emotions are oriented by moral emotion theory to explain how affective evaluation guides our response towards distance or transgression.

In this way, together, these aspects helped interpret the taboo patterns not merely as plot devices but also mechanisms for perceiving emotions, behaviors, at the level of structure organization, boundary representation and emotional coloring. Comparing these multiple aspects in the above several stories, some regularities can be found among all of them; therefore, this is not due to individual differences but more so a stable cultural belief.

### 3.4 Procedure

There are a total of four steps in this step-by-step description. Firstly, all of the selected stories were carefully read and summarized to extract the plot and determine which parts contain taboos. In the second stage, instances of prohibition, concealment or limited interaction were coded and grouped by type (such as visual taboos, spatial taboos and verbal taboos). In the third phase, after comparing the narrative consequences of transgression - such as separation, transformation, or permanent loss - among different texts to explore common emotional and relational experiences. Finally, these patterns are explained in combination with cultural-historical background, and thus, a developmental model of changes in people’s perception of the human-nature boundary across various narrative levels is built. The above process achieved high fidelity to the text and some extent of comparative analysis among cases.

To ensure the analytic transparency of taboo motifs, a stepwise coding procedure was used for operationalization. Initially, all narratives were read in their entirety to determine which explicitly prohibited or restricted perception, movement, contact, and so on. These parts were marked but not interpreted. Second, each marked segment was treated as a discrete coding unit, defined as a bounded narrative episode in which a restriction is introduced, enacted or violated. The third is that the units are divided into five types: P for perception, S for space, B for body, T for time, and R for relationship.

### 3.5 Trustworthiness and reflexivity

Given that qualitative research is subject to interpretation, rigorous analysis has been strengthened from three aspects. To enhance the interpretive quality of qualitative analysis, several strategies were adopted in the course of research design and data collection and processing.

First, interpretations were based on explicit textual evidence rather than subjective feelings, and patterns were only identified when they appeared consistently in multiple stories.

Second, the coding scheme was revised repeatedly to ensure conceptual clarity and internal consistency.

Third, theoretical claims were validated against established scholarship in folklore studies and cultural psychology studies to avoid unfounded generalizations.

The aim was not to provide a definitive standard for reading for everyone, but rather to offer an open, theoretically supported reference that can be reproduced under the same conditions.

## 4 Results

### 4.1 Recurrent patterns in taboo episodes

When examined closely at the level of encoded materials, prohibitions do not occur randomly within the story. As shown in Tables 1, 2, taboo incidents are more likely to happen at the same point in the story, generally after a period of relatively stable family situation for the human character and his non-human wife. The narration of daily life uses an ordinary style; share houses and meals, prepare food, work hard, raise children, etc., followed by a short remark: do not look, do not follow, do not open the container, do not ask.

In general, they are not long or noticeable rules. The resulting damage exceeds what are the actual reasons. At first glance, and even in some tiny action of curiosity, such a break will occur continuously throughout the text. Non-human partners disappear; they return to other dimensions or are completely lost forever. In fact, they can be regarded as boundaries that shape the development direction of narratives in this way.

Although there are many works that have used the same structure of “Union-Prosperity-Prohibition-Violence-Separation,” they differ in emotion coloring and thinking methods. Narrative-psychoanalytically speaking, there exists an organized arrangement of experiences through such disruptions that cause expectations to be overturned and thus create significance. When comparing the episodes according to the coding framework, some discrepancies will arise among them and each one belongs to multiple categories. As mentioned above, the following will introduce each of them along with those stages shown in Tables 1, 2.

### 4.2 Narratives of relative intimacy: the intimate stage

The first cluster, which represents the Intimate stage, has a higher frequency in the heterogeneous wife corpus (Table 1). All six cases have that non-human spouse did not cause any initial conflict at first, so they would become regular members of the family after becoming spouses. She weaves cloth, cooks meals for her own family, gives birth to children and so on. None of these stories indicate that there should be no interaction at the beginning.

Given this background, most of the prohibitions are perceived ones. Of the six coded episodes, five request that viewers do not watch; four ask whether there is a hidden body and if one should be revealed. They are more functions for maintaining private interests than indications of danger. They are, to a certain extent, weakly regulated boundary symbols which maintain categorial division without blocking closeness. Physical exclusion or coercive restrictions are mostly absent.

Generally speaking, the violation arises from curiosity or negligence and not with intent. Husbands look inside the forbidden zone, observe changes or accidentally expose his spouse’s real status. These events are shown to be unintentional errors rather than intentional transgressions. Revelations themselves are not portrayed in an intimidating way; they tend to be quiet.

As shown in Table 1 and others, these are all consistent. After the violation, the wife tends to leave alone in the sky, on the mountains or among animals. Separation is final but not violent. Mainly expresses a sense of sadness, remorse and depression among others. Several stories directly illustrate the husband’s regret after the loss.

This type of pattern suggests that proximal to non-human remain affects emotion rather than fear.

Generally speaking, at this stage, human beings live with nonhuman objects in their daily lives and play different social roles within society. There are boundaries, which may be porous; emotion is driven more by intimacy than by exclusion.

### 4.3 Narratives of uneasy coexistence: the transitional stage

The second cluster belongs to the Transitions period, and most of its members are husbands’ stories (Table 2). Here the possibility of the relationship still exists, but there is more uncertainty at the beginning. The way that concealment works in narration is usually represented by secret rooms, locked objects and clearly designated restricted areas.

Compared to the intimate cases, the prohibitions do not have a sense of being personal wishes but rather seem to be compulsory warnings. This shift implies an increase in boundary control within the underlying cultural schema. Curiosity has a higher risk. After removing the restriction and discovering their true identities among partners often leads to confusion or shock. Description is more unusual and frightening, not familiar.

These separations are neither accidental nor deliberate, rather they have their own reasons. Before breaking the taboo, it seems unstable due to lack of persistence in their connection; as though they are stuck somewhere temporary and not meant to last long. Emotions in these stories are generally anxiety, unease and ambivalence rather than sadness alone. A middle-type of behavior pattern that exhibits a balance between attraction and hesitation.

Therefore, they are in the intermediate position. Cross-domain contact is still possible; however, strict control should be enforced, and it will be more susceptible to interference. Proximity and distance are mutually conflicting, reflecting the instability of boundary cognition.

### 4.4 Narratives of distance and rejection: the defensive stage

The last cluster corresponds to the Defensive stage and makes up most of the remaining husband narratives in Table 2. In the story, from the beginning it is marked that the non-human figure is potentially threatening and incompatible with human society. The prohibitions function less as requests and more as guarantees.

Boundary regulation involves perception, and it not only includes the relational and bodily limitations but also has prohibitions on pursuing others, warnings about marriage, or collective acts to prevent further interaction. The form of violation may not only be simple, but also more active and confrontational, such as involving multiple people including the family or community members.

The descriptions of the scenes when revelations happen are more detailed than those in the previous sections. An exposed body may seem overly animalistic or uncanny, and it can also evoke fear, disgust, or shock. The emotional states that appear in these situations include tension, distrust, and disgust, but not regret.

Narrative outcomes are accordingly serious. Multiple endings involve expulsions, separations by force, and even deaths of non-human spouses. Separation is not a kind of loss, but it has been protected by necessity; coexistence is undesirable or dangerous.

Emotional response functions as a mechanism of exclusion and reinforces the boundary rigidity.

These narratives show that people have developed a defensive way of thinking about the boundaries, actively defending their distance from non-human others.

### 4.5 From coded patterns to stage differentiation

When the two corpora are combined, there is an alteration in the coded episodes. The same type of structuring, which is prohibition plus violation, has not altered; it just differs in terms of emotional or interpersonal setting around. Intimate narratives still maintain an accepted form of cohabitation in rule. In transitional cases, it regulates an already unstable relationship. In defensive cases, its function is generally to avoid or end contact.

Patterns are analyzed in these three directions: Narrative Structure; Boundary Representation; Emotional Orientation. They correspond to the theoretical framework of narrative psychology, cultural schema theory, and moral emotion theory. Therefore, the sequence of prohibition, violation, emotional response and outcome should be regarded as a system in motion on multiple planes rather than discrete analysis units.

This development is not based on a single case, instead, many cases have been compared. The analysis in Tables 1, 2 therefore provide the empirical basis for the three-stage differentiation presented in the following section. These stages do not refer to specific historical periods but instead describe the recurring situation of how people’s perceptions of boundaries with other living beings have been carried out, discussed and maintained.

## 5 Discussion analysis

### 5.1 From narrative patterns to psychological orientations

The ban on heterogenous marriage stories in this study originated from a simple observation that prohibitions in heterogeneous Marriage tales often occur at similar times and have uneven impacts. What at first seem to be minor rules - do not look, do not open, do not follow - repeatedly determine whether intimacy can be maintained or must come to an end. Read side by side, these episodes show that these are not isolated motifs but patterned ways of imagining contact with the non-human world.

**Figure 1:**
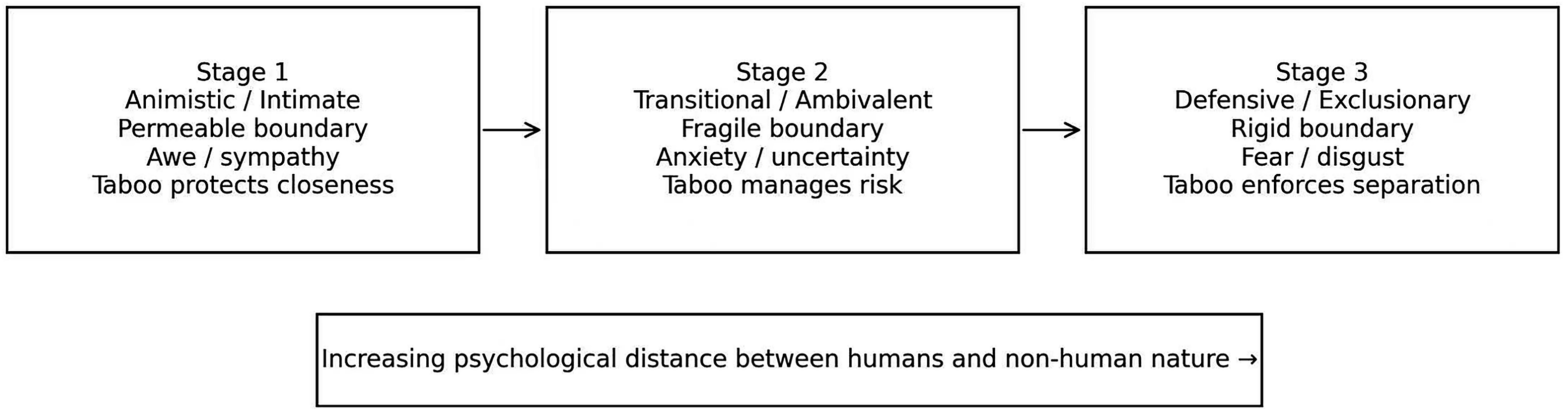
A three-stage psychological model of human-non-human boundary perception.

By comparing recurrent prohibitions, emotional reactions and separation outcomes in the stories, three relatively stable configurations emerged - intimate, transitional and defensive, which together form a stage-based psychological model of boundary cognition (Figure 1). These configurations correspond to distinct modes of boundary cognition, emerging from the interaction of narrative structure, cultural schema, and moral emotion.

In addition to regarding the forms presented in works of art as literary patterns, it is also possible that such ways of presenting information result from certain types of psychological states. Therefore, the three configurations are not presented as historical stages or fixed types. They can be considered recurring modes of assessment: relatively stable states in which proximity to alterity is felt, judged, and regulated within a cultural environment. In this sense, narrative traditions can be viewed as carriers of shared cognitive–affective schemas that organise how human–nonhuman relations are perceived.

Recently, the eco-cultural complexes have been portrayed as not only reshaping people’s cognition of nature but also affecting their emotions towards it (Kitayama and Salvador, 2024). The boundary configurations identified in these narratives can thus be interpreted as narrative expressions of culturally stabilized relational orientations toward the non-human world.

### 5.2 Intimate orientation: attachment and affiliative value

In the stories grouped as Intimate, coexistence unfolds almost matter-of-factly. Non-human spouses cook, weave, raise children, and participate in everyday routines. There is no initial indication that the relationship is essentially inappropriate. The prohibition frequently lacks the deterrent force of a threat and instead appears as a request for privacy. When it is damaged, the result is a separation, but the emotional color is quiet - more regretful than alarmed. This pattern reflects that boundary transgression does not immediately trigger defensive reactions but instead leads to affective loss.

What is outstanding here is not danger but attachment. The departing spouse often says affectionate words, while the human partner feels a loss rather than relief. The non-human body, even if it is an animal or otherworldly beings, is not typically described as grotesque. There is a boundary, but it does not require rejection yet.

Such emotional closeness is similar to the mechanism described in environmental psychology, and empathy and emotional response to nature are associated with care and protection behavior. Empirical studies show that a sense of empathy for nature can help foster prosocial and responsible values towards other living beings in people’s minds (Wang et al., 2025). From the perspective of moral emotion theory, such narratives are characterized by affiliative emotions that sustain proximity.

The concept of biophilic attachment proposed by Kellert can also serve as a reference. Accordingly, intimate narrative can be understood as a diagram structure: in this structure, the boundary remains permeable, and the emotional orientation supports the coexistence relationship.

### 5.3 Transitional orientation: uncertainty and anxiety

The moods change dramatically in the Transitional cases. Union is still possible, but it appears to be conditional. Accumulation of secrets. The doors are shut. Do not open the containers. The prohibitions are different from warnings in their lack of personalization and greater caution. Curiosity is no longer perceived.

In other words, at that time they were more hesitant or shocked than sad. Tension has already existed in their relationship before violating the rules, and it seems like they do not have any foundation of trust to start from. Intimacy is conceivable, but barely so.

This ambivalence can be seen as the coexistence of competing interpretive frames. Cultural meaning systems are not always perfect wholes, but may include partially integrated or even contradictory schemata. According to the cultural cognition theory put forward by Strauss and Quinn, there may be an unstable state or a mixed emotions in this tension. From a schema perspective, this stage reflects tension between competing boundary classifications.

Recent work on mental models of the human-nature relationship is also moving in that direction. Different groups of people may have diverse beliefs due to various reasons, such as being more biocentric or more anthropogenic; therefore, their attitudes and behaviors towards environmental problems will also differ (Kim and Coley, 2025). Thus, Transitional narratives can be interpreted as unstable schema configurations in which attraction and caution coexist, producing ambivalent emotional orientations.

### 5.4 Defensive orientation: distance and exclusion

In the defensive Configuration, the terms of engagement have changed again. The non-human figure is more explicitly marked as incompatible or dangerous. Prohibitions are actually safeguards. Boundary becomes explicit, rigid, and often socially reinforced. Thus, distance is not accidental but normatively required.

Therefore, the change is considered to be due to appraisal. Based on the framework of Lazarus, it is believed that the core part of evaluation determining whether a situation is judged as a threat is included. When the non-human other is interpreted primarily as a source of danger, emotions of avoidance and withdrawal are credible adaptive responses (Smith and Lazarus, 1990). These narratives are dominated by moral emotions such as fear and disgust, which function to enforce categorical separation.

Contemporary research on human-nature relations has begun to record this phenomenon systematically. There is also a kind of phobia called “biophobia,” which refers to fear, disgust or rejection of nature, that has gradually gained attention in psychological and environmental research; this will cause some people to be uncomfortable, such as avoiding natural environments and having a weak connection with them (Jensen et al., 2025).

Accordingly, Defensive narratives represent schema configurations in which boundaries are rigid and emotional orientation is structured by aversion and exclusion.

### 5.5 Narrative traditions as cultural schemas

The three kinds of phenomena in this paper suggest that besides specific events, traditional narratives are also culturally shared cognitive Frameworks that organise people’s perception and interpretation of the interaction between humans and non-humans. In this interpretation, stories are not only descriptions but also carry mental models that guide attention, emotion, and prediction about the non-human world. They can be understood as narrative expressions of cultural schemas.

Similar taboo structures in European swan maiden and animal bridegroom tales suggest broader cross-cultural relevance. Such boundary configurations may reflect generalizable patterns of human–nonhuman relational cognition.

Repertory structures may stabilize shared interpretive patterns, and the theory is commonly referred to in research on narrative psychology. The idea of the recently produced works is that narrative serves as a tool for cognitive prediction, organization, and adaptation at both individual and group levels in relation to socially relevant situations. In recent research on narrations has been presented as one form of active inference; that is, a kind of prediction process involving social cognition and adaptive response for complex situations (Bouizegarene et al., 2024). Thus, repeated narrative patterns may function as predictive models for evaluating boundary-crossing situations.

Strauss and Quinn (1997) have discovered that the community of people has a kind of stable cognitive cultural sense structure for this shared story, while the theory of cognitive cultures holds that such meaning patterns at higher levels predict an individual’s ability to understand their experiences across different times. Intimate, Transitional, and Defensive configurations can therefore be understood as schema families.

Emotions also seem to be interwoven within these schema structures rather than added later. Based on Ahmed’s work in the cultural politics of emotion, it can be inferred that affective patterns spread through narrative traditions and guide individuals to have specific relational orientations, thereby promoting culturally patterned responses to perceived nearness or distance (Ahmed, 2004). The emotional tendencies of the three states, attachment, unease, and aversion, are thus essential components of the schema.

In this way, taboo motifs operate not only as narrative devices but as mechanisms of boundary cognition, linking cultural schema, narrative structure, and emotional evaluation into a unified process.

## 6 Conclusion and implications

These patterns are not merely narrative regularities but reflect culturally structured cognitive–affective orientations toward the non-human world. Based on this interpretation, the following are a few implications.

First of all, based on the present findings, it can be concluded that the Heterogeneous Marriage Tales are not only symbolic representations of belief systems or aesthetic narrative conventions. They operate as structured models that repeatedly stage processes of approach, hesitation, and retreat in human–nonhuman relations. In this sense, they can be interpreted as narrative expressions of cultural schemas that guide expectations about relational boundaries. Through recurring narrative configurations of approach, hesitates, and then retreats, these stories offer recognizable models for the development of other relationships with non-human objects. Thus, folklore can be considered both cultural heritage and a psychological resource for relation expectation.

Secondly, the three schemes proposed here - Intimate, Transitional and Defensive - may not be seen as distinct historical phases, but rather as three kinds of orientation that occur repeatedly in a cultural repository. These configurations represent alternative modes of boundary cognition that may be activated under different contextual conditions. In this case, the narrative tradition not only represents people’s perceptions of nature but also constructs such perceptions through preset evaluation systems. From a cultural–psychological perspective, these patterns can be understood as long-term cognitive–affective frameworks that integrate schemas, narrative structuring, and emotional evaluation.

Thus, rather than assuming a fixed boundary between humans and the non-human world, these narratives demonstrate that such boundaries are continuously negotiated and redefined through shared cultural forms. This extends existing research on narrative identity and schema theory by highlighting the role of stories as regulators of emotional distance from non-human life.

In addition, these phenomena also show that folkloric materials may be subjects of study in cultural and environmental Psychology. Literary and oral traditions have maintained long-term records of how communities have envisioned coexisting with other forms of life. By contrasting with other materials, some patterns in people’s emotional reactions and cognitive states cannot be discovered via existing questionnaires yet. Integrating narrative analysis and psychological theory may thus expand the methodological toolkit for exploring human-nature relationships.

Lastly, these findings can be further situated within broader discussions of collective responses to environmental changes. The taboo structure identified in this study can be interpreted as a cultural coding strategy to regulate the degree of human exposure to uncertain or potentially destructive environments. Similarly, the emotional structure observed in this study - from intimacy, transition to defensiveness - reflects the collective emotional pattern accompanied by ecological uncertainty. The repeated separation ending can be understood as a symbolic solution to the boundary rupture, reflecting the environmental disturbance experience characterized by loss and breakdown. In this sense, such narratives provide a form in which human collective experiences of Earth systems change and related collective human trauma can be organised, remembered, and meaningfully interpreted. Different from directly giving answers, this paper offers an analytical theory system to reveal the laws of operation for connection among cultural imagination and psychological reactions. In the future, other traditions or fields can be selected for research to see if there is a similar boundary configuration in other cultures.

There are still some deficiencies. Based on a limited corpus and through interpretive method, the current research will bring forward some typical characteristics but ignore others as well. Without such materials, we cannot explore whether these narrative patterns still influence people today. Future research can further explore how such patterns - emotional structures can continue to affect people’s cognition and behavior in the context of environmental change through cross-cultural traditional comparisons or combining narrative analysis with empirical methods.

## Data Availability

The original contributions presented in the study are included in the article/supplementary material, further inquiries can be directed to the corresponding author.
